# *Saccharomyces cerevisiae* and *S. kudriavzevii* Synthetic Wine Fermentation Performance Dissected by Predictive Modeling

**DOI:** 10.3389/fmicb.2018.00088

**Published:** 2018-02-02

**Authors:** David Henriques, Javier Alonso-del-Real, Amparo Querol, Eva Balsa-Canto

**Affiliations:** ^1^(Bio)process Engineering Group, IIM-CSIC, Vigo, Spain; ^2^Grupo de Biología de Sistemas en Levaduras de Interés Biotecnológico, IATA-CSIC, Valencia, Spain

**Keywords:** *Saccharomyces* species, temperature, wine fermentation, dynamic modeling, parameter estimation, cross-validation

## Abstract

Wineries face unprecedented challenges due to new market demands and climate change effects on wine quality. New yeast starters including non-conventional *Saccharomyces* species, such as *S. kudriavzevii*, may contribute to deal with some of these challenges. The design of new fermentations using non-conventional yeasts requires an improved understanding of the physiology and metabolism of these cells. Dynamic modeling brings the potential of exploring the most relevant mechanisms and designing optimal processes more systematically. In this work we explore mechanisms by means of a model selection, reduction and cross-validation pipeline which enables to dissect the most relevant fermentation features for the species under consideration, *Saccharomyces cerevisiae* T73 and *Saccharomyces kudriavzevii* CR85. The pipeline involved the comparison of a collection of models which incorporate several alternative mechanisms with emphasis on the inhibitory effects due to temperature and ethanol. We focused on defining a minimal model with the minimum number of parameters, to maximize the identifiability and the quality of cross-validation. The selected model was then used to highlight differences in behavior between species. The analysis of model parameters would indicate that the specific growth rate and the transport of hexoses at initial times are higher for *S. cervisiae* T73 while *S. kudriavzevii* CR85 diverts more flux for glycerol production and cellular maintenance. As a result, the fermentations with *S. kudriavzevii* CR85 are typically slower; produce less ethanol but higher glycerol. Finally, we also explored optimal initial inoculation and process temperature to find the best compromise between final product characteristics and fermentation duration. Results reveal that the production of glycerol is distinctive in *S. kudriavzevii* CR85, it was not possible to achieve the same production of glycerol with *S. cervisiae* T73 in any of the conditions tested. This result brings the idea that the optimal design of mixed cultures may have an enormous potential for the improvement of final wine quality.

## Introduction

Wine is obtained through the fermentation of grape must, a complex media composed by a rich blend of amino acids, sugars, organic acids, vitamins and the list goes on. Modern wine industry selects specific yeasts to inoculate the grape must and to perform controlled fermentations. This approach reduces the risk of wine contamination while increasing reproducibility and enabling the production of wines with specific aromas or other compounds of interest. Selecting appropriate yeast species may contribute to face the challenges brought by climate change, but also to increase the variety and quality of wines, as consumers and market demand.

Most of the commercial yeasts belong to the *Saccharomyces cerevisiae* species, therefore being the most frequently used in wine making, as well as the most studied species. However, other yeasts, such as non-*Saccharomyces* species, have shown their potential to solve the new challenges of the wine making industry (Ciani et al., 2016; Pérez-Torrado et al., 2017). Interestingly, species of the *Saccharomyces* genus, such as *Saccharomyces kudriavzevii*, exhibit promising physiological properties. *S. kudriavzevii* ferments at lower temperatures (Salvadó et al., 2011), produces less ethanol and more glycerol (Oliveira et al., 2014; Pérez-Torrado et al., 2016) with no increase in the acetic acid levels in wine (Alonso-del Real et al., 2017), and generates a higher content of aromatic superior alcohols (Stribny et al., 2016).

Temperature is one of the most important parameters affecting the duration and rate of alcoholic fermentation and final wine quality. Many wine makers prefer low-temperature fermentations (10–15°C) for the production of white and “rosé”. Wines produced at low temperatures keep volatile aroma compounds more efficiently; therefore, showing better sensory attributes. However, the performance of *S. cerevisiae* at low temperatures decreases, due to growth rate reduction and an increased risk of stuck and sluggish fermentations (López-Malo et al., 2013). Recent studies have confirmed that the cryophilic yeast *S. kudriavzevii* performs better than *S. cerevisiae* at low temperature, thus being an appealing alternative for cold fermentations (Tronchoni et al., 2012). Additionally, *S. kudriavzevii* produces less alcohol than *S. cerevisiae* offering a means to handle the rising sugar content in grape must (Alonso-del Real et al., 2017). Nevertheless, the feasibility of using non-conventional yeasts, such as *S. kudriavzevii*, at the industry, requires an improved understanding of the physiology and metabolism of these cells.

Dynamic modeling brings the potential of exploring the most relevant mechanisms underlying fermentation performance by different species but also the possibility of designing optimal operating conditions more systematically (Banga et al., 2005; Pizarro et al., 2007). The modeling of wine fermentation has received substantial attention. Depending on their aim, available models can be classified into macroscopic kinetic or intracellular metabolic. Macroscopic kinetic models are focused on biomass growth and external metabolites. They require the definition of kinetic rates as functions of the intervening species concentrations. Metabolic models consider cellular metabolic pathways which are defined in terms of fluxes; an optimization based approach is then used to compute metabolic flux profiles compatible with the measured dynamics of biomass growth.

The pioneering works by Boulton (1980) or Caro et al. (1991) adopted the macroscopic scale modeling approach. Subsequently several works focused on the efficiency of *S. cerevisiae* to transform glucose to ethanol within a range of temperatures around that corresponding to the optimal growth (see, for example, the review by Marín, 1999 and the works cited therein). More recently, Cramer et al. (2002), Malherbe et al. (2004), and Coleman et al. (2007) also adopted the macroscopic scale modeling approach to address the role of assimilable nitrogen in ethanol and CO_2_ production. Agosin and collaborators considered the cellular metabolism within a dynamic flux balance modeling framework (Sainz et al., 2003; Varela et al., 2004; Pizarro et al., 2007; Vargas et al., 2011). These models reproduced the measured dynamics of biomass growth, substrates uptake as well as ethanol and glycerol production. Alternatively Malherbe et al. (2004) or David et al. (2010) adopted an intermediate strategy that couples the kinetic modeling of external metabolites with some intracellular mechanisms. Their focus is on the role of nitrogen.

In this work we adopt the later strategy to model cold fermentations mediated by non-conventional *Saccharomyces* species. For this purpose we implemented an experimental-modeling pipeline. The experimental pipeline is based in micro-vinifications where small-scale wine fermentations are undertaken at different controlled conditions while monitoring growth rate and a number of critical extracellular metabolites (glucose, fructose, ethanol, glycerol, and acetic acid).

The modeling pipeline is based on model selection, reduction, ensemble modeling and cross-validation. Several candidate models -which account for different biomass growth, transport and inhibitory mechanisms found in the literature- are compared attending to their properties, basically identifiability and robustness in cross-validation. In this respect, we focused on defining a minimal model with the minimum number of parameters to guarantee structural identifiability, i.e. the possibility of uniquely reconciling the model with the data while iteratively improving practical identifiability (Chis et al., 2016). For the most successful models we implemented an ensemble modeling strategy so as to maximize their robustness, i.e., to minimize the uncertainty of their predictions. The results from the obtained models are discussed in a quantitative manner and ensemble of models is used to devise robustified predictions for processing conditions (initial inoculation and temperature) so as to achieve a better compromise between alcohol and glycerol production.

The selected model accounts for the transport of hexoses (glucose and fructose) and their transformation into fructose 6-phosphate (F6P); the F6P is then directed to produce both ethanol, acetic acid and glycerol. The model considers the temperature effects in the cells specific growth rate; but also temperature and ethanol as inhibitors of the transport of hexoses. As a result it can be used to design cold wine fermentations to optimize final product quality.

Finally, we show, by means of cross-validation, that using an ensemble approach delivers more robust solutions than using a single model approach, thus rendering the ensemble models useful to explain the differences in fermentation performance between the species of interest and to design novel wine-making processes.

## Materials and methods

### Experimental methods

#### Strains

We considered two different *Saccharomyces* strains. We chose a commercial strain, T73 (Lalvin T73 from Lallemand Montreal, Canada), as our wine *S. cerevisiae* representative, and *S. kudriavzevii* strain CR85, a natural isolate from oak tree bark in Agudo, Ciudad Real, Spain. Throughout the rest of the this paper these strains will be referred to as SKCR85 and SCT73, respectively.

#### Synthetic must fermentations

All fermentations were performed in 3*x* replicates in 250 mL flasks that contained 200 mL of synthetic must (SM) miming a standard natural must which is frequently used in microvinification experiments (Rossignol et al., 2003). This medium contains 100 g/L glucose and 100 g/L of fructose, mineral salts (750 mg/L KH_2_PO_4_, 500 mg/L K_2_SO_4_, 250 mg/L MgSO_4_.7H_2_O, 155 mg/L CaCl_2_.2H_2_O, 200 mg/L NaCl, 4 mg/L MnSO_4_.H_2_O, 4 mg/L ZnSO_4_, 1 mg/L CuSO_4_.5H_2_O, 1 mg/L KI, 0.4 mg/L CoCl_2_.6H_2_O, 1 mg/L H_3_BO_3_, 1 mg/L NaMoO_4_.2H_2_O), vitamins (20 mg/L myo-inositol, 2 mg/L nicotinic acid, 1.5 mg/L calcium panthothenate, 0.25 mg/L thiamine HCl, 0.25 mg/L pyridoxine HCl, 0.003 mg/L biotin), 300 mg/L of assimilable nitrogen (ammoniacal nitrogen and α-amino nitrogen) provided by a mixture of 19 amino acids (612.6 mg/L L-proline, 505.3 mg/L L-glutamine, 374.4 mg/L L-arginine, 179.3 mg/L L-tryptophan, 145.3 mg/L L-alanine, 120.4 mg/L L-glutamic acid, 78.5 mg/L L-serine, 759.2 mg/L L-threonine, 48.4 mg/L L-leucine, 44.5 mg/L L-aspartic acid, 44.5 mg/L L-valine, 37.9 mg/L L-phenylalanine, 32.7 mg/L L-isoleucine, 32.7 mg/L L-histidine, 31.4 mg/L L-methionine, 18.3 mg/L L-tyrosine, 18.3 mg/L L-glycine, 17.0 mg/L L-lysine, and 13.1 mg/L L-cysteine) corresponding to 180 mg nitrogen and 460 mg/L ammonium chloride (corresponding to 120 mg nitrogen). The pH was buffered at 3.3 with NaOH.

We monitored the growth of each strain in monocultures under the same conditions. Overnight precultures were grown in YPD medium at 25°C. Afterwards must was inoculated with the corresponding yeast strain to reach an initial concentration of 10^6^ cells/mL, and was incubated at a fixed temperature (8, 12, 20, or 25°C) with agitation at 100 RPMs during fermentation.

Cell samples were collected at several time points during fermentation. Growth curves were obtained by considering cell density calculated from cell counting in a Neubauer chamber (Alonso-del Real et al., 2017). Müller valves were used to monitor fermentation stage through weight loss, until it reached a constant weight, when it was considered to be over. At this point, samples of supernatant were kept at −20°C for further analyses.

#### High performance liquid chromatography

Residual sugars (glucose and fructose), glycerol, ethanol and acetic acid from the fermentation end point samples were determined by HPLC (Thermo Fisher Scientific, Waltham, MA. USA) using a refraction index detector and a HyperREZTM XP Carbohydrate H+ 8μ*m* column (Thermo Fisher Scientific) equipped with a HyperREZTM XP Carbohydrate Guard (Thermo Fisher Scientific). Samples were diluted to maintain our target compounds within the allowed range of detection, filtered through a 0.22 μM nylon filter (Symta, Madrid, Spain) and injected in duplicate. The analysis conditions were: eluent, 1.5 μM of H_2_SO_4_; 0.6 mL/min flux and a 50°C oven temperature.

### Theoretical methods: the modeling pipeline

Modeling was approached from a systems identification perspective including the following steps: formulation of candidate models, multi-experiment parameter estimation, model selection and reduction, ensemble modeling and cross-validation.

#### Formulation of candidate models

We formulated several candidate models which account for the relevant process variables (biomass growth, sugars, ethanol, glycerol, acetate) based on different mechanisms described in literature. All candidate models consist of a set of ordinary differential equations whose solution depends on the given initial conditions, process temperature and the value of a number of unknown parameters.

#### Parameter estimation

The aim of parameter estimation is to compute the unknown parameters - growth related constants and kinetic parameters - that minimize the distance among data and model predictions. The maximum-likelihood principle yields an appropriate measure of such distance (Walter and Pronzato, 1997):

(1) $$J_{mc}(\theta )=\sum_{k=1}^{n_{exp}}\sum_{j=1}^{n_{obs}}\sum_{i=1}^{n_{st}}{\left(\frac{y_{k,j,i}(\theta )-y_{k,j,i}^{m}}{\sigma_{k,j,i}}\right)}^{2},$$

where *n*_*exp*_, *n*_*obs*_ and *n*_*st*_ are, respectively, the number of experiments, observables, and sampling times while σ_*k, j, i*_ represents the standard deviation of the measured data as obtained from the experimental replicates. $y_{j}^{m}$ represents each of the measured quantities, *X*^*m*^ and *C*^*m*^ in our case, and *y*_*j*_(***θ***) corresponds to model predicted values, *X* and *C*.

Parameters are estimated by solving a nonlinear optimization problem where the aim is to find the unknown parameter values (***θ***) to minimize *J*_*mc*_(***θ***), subject to the system dynamics—the model—and parameter bounds (Vilas et al., 2018).

#### Model selection and reduction

Models were compared, first, attending to their capabilities to fit the experimental data. Since models with a larger number of parameters tend to provide better fits, which may lead to over-fitting, we also considered the number of parameters in our comparison. For this purpose we used the Akaike information criterion (AIC) defined as follows (Burnham and Anderson, 2002):

(2) $$AIC=2n_{p}+n_{d}\cdot ln(J),$$

where *n*_*p*_ is the number of unknown adjustable parameters, *n*_*d*_ the number of data.

We started with most complex candidate models after data fitting less influencing parameters were iteratively removed from the model following an AIC based strategy. Parameters were removed as long as the AIC was reduced, otherwise, the reduced model was rejected. The decision tree used to simplify the models is detailed in the Supplementary Information.

The most promising candidate models were further compared in terms of their associated uncertainty in cross-validation.

##### Uncertainty analysis

In practice, the value of the parameters ***θ*** compatible with noisy experimental data is not unique, i.e., parameters are affected by some uncertainty. The consequence of significant parametric uncertainty is that the model may not be able to predict scenarios other than those used in parameter estimation.

To measure the actual model predictive capabilities, the model is usually given a dataset of known data on which training is run (training dataset), and a dataset of unknown data against which the model is tested (testing dataset). The training dataset regards the data used for parameter estimation; while the testing dataset is obtained under untrained experimental conditions (for example, a different process temperature).

To account for model uncertainty we used an **ensemble approach**. To derive the ensemble we apply the bootstrap smoothing technique, also known as bootstrap aggregation (the Bagging method) in the prediction literature (Breiman, 1996; Bühlmann, 2012). The bagging method is a well established and effective ensemble model/model averaging device that reduces variability of unstable estimators or classifiers (Bühlmann and Yu, 2002). The underlying idea is to consider a family of models with different parameter values $\Theta ={\left[\theta_{1}\ldots \theta_{N}\right]}^{T}$ compatible with the training data *y*^*m*^, when using the model to predict untested experimental setups. The matrix of parameter values **Θ** consistent with the data is obtained using *N* realizations of the data obtained by bootstrap (Efron and Tibshirani, 1988). Each data realization has the same size of the complete data-set but it is constructed by sampling uniformly from all replicates (3 biological replicates per sampling time). Therefore at each bootstrap iteration, a given replicate has an approximate chance of 37% from being left out, while others might appear several times (2,3,…) in a given instance of the bootstrap. The family of solutions, **Θ**, is then used to make *N* predictions (dynamic simulations) about a given experimental scenario. The median of the simulated trajectories regards the model prediction while the distribution of the individual solutions at a given sampling time provide a measure of the uncertainty of the model.

**Cross-validation** In order to test the modeling predictions under untested conditions we apply out-of-sample cross-validation (Elsner and Schmertmann, 1994; Tashman, 2000). To compute the ensemble of predictions for each tested temperature for which we have experimental data, i.e., we omitted the experimental data for each temperature and computed an ensemble model for each scenario (Henriques et al., 2017). Finally we used the obtained models to compute a median solution for each temperature and assess the quality of the solutions using the root mean square error metric:

(3) $$\text{RMSE}(\theta )=\sqrt{\frac{\sum_{k=1}^{n_{exp}}\sum_{j=1}^{n_{obs}}\sum_{i=1}^{n_{st}}{\left(y_{k,j,i}(\theta )-y_{k,j,i}^{m}\right)}^{2}}{N_{Data}}}$$

where *N*_*Data*_ corresponds to the number of data points used for training and testing. The comparison between the root mean square error in training and in testing gives a measure of the capabilities of the model to predict untested conditions. As it is defined, the RMSE is scale dependent. To provide a normalized value (NRMSE) it is possible to divide by the maximum measurement for each species.

Model selection can be done by comparing the NRMSE as obtained for the training and testing conditions. The lower the NRMSE values the better the model.

#### Numerical tools

To automatize the modeling pipeline we used the AMIGO2 toolbox (Balsa-Canto et al., 2016). AMIGO2 is a MATLAB based software tool focused on parametric model identification and optimization, including sensitivity and identifiability analyses. It offers a suite of numerical methods for both simulation and optimization. From the available options we selected CVODES (Hindmarsh et al., 2005) to solve the model equations, and *Enhanced Scatter Search* (eSS, Egea et al., 2009), to find the optimal parameter values in reasonable time.

The ensemble model generation and cross-validation procedures are computationally intensive. However, since each parameter estimation instance in the ensemble is a completely independent task, we were able to solve this problem in less than a day using 60 CPU cores on a Linux cluster. These tasks were automated with the help of bash scripts and the Open Grid Scheduler. All the scripts necessary to reproduce the results are distributed as part of the Supplementary Materials.

## Results and discussion

### Formulation of candidate models

This work seeks a minimal yet predictive model to describe the fermentation processes mediated by two different *Saccharomyces* species under a range of cold temperature processing conditions.

Previous modeling efforts focused on the efficiency of *S. cerevisiae* to transform glucose to ethanol within a range of temperatures around that corresponding to the optimal growth (see, for example, the review by Marín, 1999 and the works cited therein). Later, Cramer et al. (2002), Malherbe et al. (2004), or David et al. (2010) proposed oenological models which account for the role of nitrogen sources in sluggish or stuck fermentations.

However, the primary motivation to use other yeasts as wine making starters is to improve final product characteristics such as enhanced glycerol content, low temperature fermentation kinetics or novel attractive aroma profiles. Unfortunately, these previous models do not include glycerol or acetic acid, and many of them do not take into account the role of the temperature, rendering them as non-valid for our purposes.

**Figure 1:**
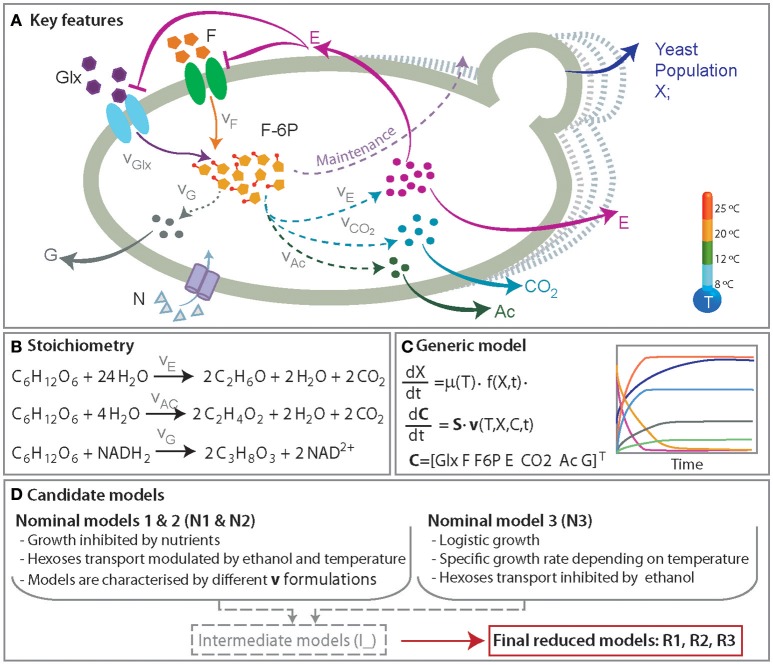
A simplified representation of the anaerobic metabolism of glucose and fructose. **(A)** Presents the key features of the process including yeast population growth and the production of ethanol (E), glycerol (G), acetic acid (Ac), and CO_2_. The roles of the temperature and ethanol production in the hexoses transport (glucose, Glx and fructose, F) and growth are also incorporated. **(B)** Presents the set of reactions. **(C)** Shows an overview of the mathematical model consisting of a set of ordinary differential equations describing the dynamics of yeast population and the relevant metabolites concentrations. **(D)** summarizes the major mechanisms included in the candidate models.

We put particular emphasis on developing a minimal model, with nice mathematical properties (i.e., identifiable) and yet comprehensive in the sense of the mechanisms involved. With this aim we formulated three candidate models, regarded as nominal models, which describe the accumulation of extracellular ethanol, glycerol, acetic acid and release of C0_2_. We also included a simplified model of glycolisis that respects mass conservation coupled to alternative growth models and the transport of hexoses. Figure 1 shows an overview of the relevant species included in the candidate models.

#### Modeling growth

We considered two different alternatives to model biomass (*X*) dynamics. On the one hand, a linear model accounting for substrate inhibition (in nominal models N1 & N2) and on the other, the Verhulst logistic model (in nominal model N3).

Nominal models N1 & N2 assume linear biomass growth being the specific growth rate modulated by glucose (υ_*tr,G*_) and fructose transport (υ_*tr,F*_):

(4) $$\dot{X}=\mu (\upsilon_{tr,G},\;\upsilon_{tr,F})\cdot \;X$$

These models account for the growth inhibition due to limited substrate. The synthetic must used in our experiments contains 300 mg/L of assimilable nitrogen which is enough for the yeast to reach its maximum fermentation rate and for no issues to arise during fermentation. Therefore assimilable nitrogen is not considered as an inhibiting substrate. However, its initial amount was considered in nominal model N1.

The logistic model (in nominal model N3) is the standard in predictive microbiology (Baranyi and Roberts, 1994) and was also used by Malherbe et al. (2004) or David et al. (2010) to model wine fermentation. The model accounts for intra-species competition for the available nutrients in such a way that the specific growth rate (μ) depends on the environmental conditions (temperature, T, in our case) and the maximum biomass (*X*_*max*_), also known as the species carrying capacity, depends on the available nutrients. The logistic model is defined as follows:

(5) $$\dot{X}=\mu_{T}(T)\cdot \;X\cdot \;(1-\frac{X}{X_{max}})$$

The specific growth rate depends on the temperature. To include this dependency with the minimum number of parameters we explored previously published data (Arroyo-López et al., 2009). In the range of temperatures of interest, μ(*T*) can be well approximated by a quadratic function.

(6) $$\mu_{T}(T)=k_{T2}\cdot \;T^{2}-k_{T1}\cdot \;T+k_{T0}$$

#### Modeling the transport of hexoses

Yeasts use several hexose transporters, which transport glucose and fructose amongst other sugars, by facilitated diffusion (Boles and Hollenberg, 1997). Although yeasts show preference for glucose (Berthels et al., 2004), glucose and fructose can be consumed simultaneously.

Hjersted et al. (2007) modeled the transport of hexoses using a Michaelis-Menten (MM) type kinetics as follows:

(7) $$\upsilon_{tr,H}=X\cdot \;\frac{k_{H}\cdot \;H}{k_{sH}+H}$$

where *k*_*H*_, refers to the transport rate; *k*_*sH*_ regards the Michaelis constant; *H* refers to the relevant hexoses (glucose and fructose) and *X* is the number of cells.

It should be noted that the transport of hexoses is a very complex process which will be affected by both temperature and ethanol. We took these effects into account by modifying the Equation 7 as follows:

(8) $$\upsilon_{tr,H}=X\cdot \;\phi_{T}\cdot \;\phi_{E}\frac{k_{H}\cdot \;H}{k_{sH}+H}$$

in such a way that we uncouple the effects of temperature (ϕ_*T*_) and ethanol(ϕ_*E*_).

We modeled the effect of temperature with a couple of empirical functions taken from the literature, ϕ_*T,A*_ and ϕ_*T,B*_, defined as follows:

(9) $$\phi_{T,A}=(a/T^{2})\cdot \;e^{-b/T}+c\cdot \;N_{0}$$

This expression, proposed by Pizarro et al. (2007), was considered in nominal model N1 and accounts, not only for the effect of temperature but also for the initial amount of assimilable nitrogen. The expression contains three parameters: *a, b* and *c* to be estimated from data; *N*_0_ regards the initial amount of assimilable nitrogen in the medium.

(10) $$\phi_{T,B}=a\cdot \;e^{-b/T}$$

where *a* regards the intensity of the temperature effect and *b* is the rate of the exponential function and *T* is the temperature. This expression, proposed by Malherbe et al. (2004) and later used by Charnomordic et al. (2010), indicates that transport increases with temperature. This increase is an overall effect resulting from the contribution of different processes: the production of different transporters with different transport affinities which may depend on temperature (Tai et al., 2007; Postmus et al., 2008) and the effect of the amount of intracellular hexoses (Teusink et al., 1998) being directed to glycolysis. ϕ_*T,B*_ was incorporated in the nominal model N2.

Finally, ethanol has been reported as a non-competitive inhibitor (Leão and Van Uden, 1982) of glucose transport. We modeled its effect as follows (Hjersted et al., 2007):

(11) $$\phi_{E}=\frac{1}{1+E/K_{Ei}}$$

where *K*_*Ei*_ defines the strength of the inhibitory effect.

It should be noted that, to guarantee structural identifiability, *k*_*H*_ and *a* can not be simultaneously estimated from experimental data, but only their product, ν_*G*_ = *k*_*H*_ · *a*.

#### Metabolic model

In order to provide a simple representation of the metabolism while avoiding over-parameterization and lack of identifiability, we assume that upon transport, glucose and fructose are rapidly metabolized into Fructose 6-Phosphate (F6P).

During alcoholic fermentation, F6P_*in*_ (which regards the concentration of F6P per cell) is metabolized to pyruvate, through a number of steps, via the glycolytic pathway. Pyruvate is then decarboxylated into acetaldehyde and finally reduced to ethanol or acetate. All these intermediate steps are lumped into the rates υ_*F*6*p*→*E*_ and υ_F6P → Ace_ first is described using irreversible Michaelis-Menten type kinetics while the later is described using mass action law. Additionally, part of the carbon flux is redirected to the glycerol pathway. Glycerol production is described with a mass action type equation. Moreover, the rate function, υ_*manteinance*_, explaining the conversion of F6P into biomass or other maintenance costs is added to account for what was not converted into Glx, G or ACE. From the former rates we are able to derive the following set of ordinary differential equations (ODEs) describing the molar concentration of the different metabolites considered:

(12) $${\text{F6P}}_{in}=\frac{\text{F6P}}{X}$$

(13) $$\upsilon_{Maintenance}=X\cdot k_{Maintenance}\cdot {\text{F6P}}_{in}$$

(14) $$\upsilon_{\text{F6P}\to \text{E}}=X\cdot k_{E}\cdot \frac{{\text{F6P}}_{in}}{k_{s,E}+{\text{F6P}}_{in}}$$

(15) $$\upsilon_{\text{F6P}\to \text{Ace}}=X\cdot k_{Ace}\cdot {\text{F6P}}_{\text{in}}$$

(16) $$\upsilon_{\text{F6P}\to \text{G}}=X\cdot k_{G}\cdot {\text{F6P}}_{\text{in}}$$

(17) $$\begin{array}{l} \dot{\text{F6P}}=\upsilon_{tr,Glx}+\upsilon_{tr,F}-\upsilon_{\text{F6P}\to \text{E}}-\upsilon_{\text{F6P}\to \text{G}}\\ \;-\upsilon_{\text{F6P}\to \text{Ace}}-\upsilon_{Maintenance} \end{array}$$

(18) $$\dot{\text{E}}=2\cdot \upsilon_{\text{F6P}\to \text{E}}$$

(19) $$\dot{\text{G}}=2\cdot \upsilon_{\text{F6P}\to \text{G}}$$

(20) $$\dot{\text{Ace}}=2\cdot \upsilon_{\text{F6P}\to \text{Ace}}$$

(21) $$\dot{\text{CO2}}=2\cdot \upsilon_{\text{F6P}\to \text{Ace}}+2\cdot \upsilon_{\text{F6P}\to \text{E}}$$

where *F*6*P*_*in*_ corresponds to the concentration of F6P per cell; *k*_*Maintenance*_, *k*_*Ace*_, *k*_*G*_ correspond to reaction rates for biomass maintenance and the production acetate and glycerol respectively; *k*_*s,E*_ is the FP6 per cell concentration at which the reaction rate is half of its maximum, *k*_*s,E*_; CO_2_ represents the concentration of carbon dioxide released when ACE and E are produced. The coefficient (2) included in Equations 19–21 accounts for the stoichiometry of the reaction as described in Figure 1.

#### Model selection and reduction

All nominal candidate models consist of 7 ordinary differential equations. However, they differ in the number of adjustable unknown parameters. The parameter estimation for each model was performed by using the total of 329 data points for both species. It should be noted that a limited number of sampling times is available for the experiments performed at 20°C. The parameter estimation of the nominal models revealed several non influencing parameters which called for model reduction. Details on the various intermediate reduced models can be found in the Supplementary Information. Table 1 presents the major characteristics of and the best fit statistics for the nominal models plus the final reduced models.

**Table 1:** Major characteristics and best fist statistics for nominal models and final reduced models.

**Model**	**Model characteristics**	**Best fit, J**	**AIC**	**#Pars**
N1	- Linear growth	6.78	335.63	31
	- Growth rate depending on the transport of hexoses			
	- Michaelis-Menten transport of hexoses			
	- ϕ_*T, A*_			
	- ϕ_*E*_			
N2	- Linear growth	5.51	313.95	35
	- Growth rate depending on the transport of hexoses			
	- Michaelis-Menten transport of hexoses			
	- ϕ_*T, B*_			
	- ϕ_*E*_			
N3	- Logistic growth	4.68	286.65	33
	- Quadratic growth rate			
	- Michaelis-Menten transport of hexoses			
	- ϕ_*T, B*_			
	- ϕ_*E*_			
R1	- Linear growth	6.92	326.52	25
	- Michaelis-Menten transport of hexoses			
	- ϕ_*T, A*_			
	- ϕ_*E*_			
R2	- Linear growth	5.73	291.58	21
	- Linear transport of hexoses			
	- ϕ_*T, B*_			
	- ϕ_*E*_			
R3	- Logistic growth	4.87	276.26	25
	- Quadratic growth rate			
	- Linear transport of hexoses			
	- ϕ_*T, B*_			
	- ϕ_*E*_			

Reduced models are better in terms of the Akaike criterion as compared to their nominal counterparts. The best model in terms of quality of fit is the nominal model N3; while models N1 and N2 based on linear growth with growth rate depending on the substrates where less successful. Note, however, that the reduced model R3 is indeed better than N3 in terms of the Akaike criterion. In R3 the Michaelis-Menten (MM) type kinetics explaining hexoses transport (Hjersted et al., 2007) was reduced to mass action kinetics. Remarkably this reduction was also needed for nominal models 1 and 2, indicating that data coming from fermentations occurring at different initial amounts of glucose and fructose are required to identify Michaelis-Menten kinetics. Similarly, in N1 the term corresponding to the initial amount of assimilable nitrogen was reduced due to lack of identifiability.

### Model ensemble and cross-validation for reduced models

**Figure 2:**
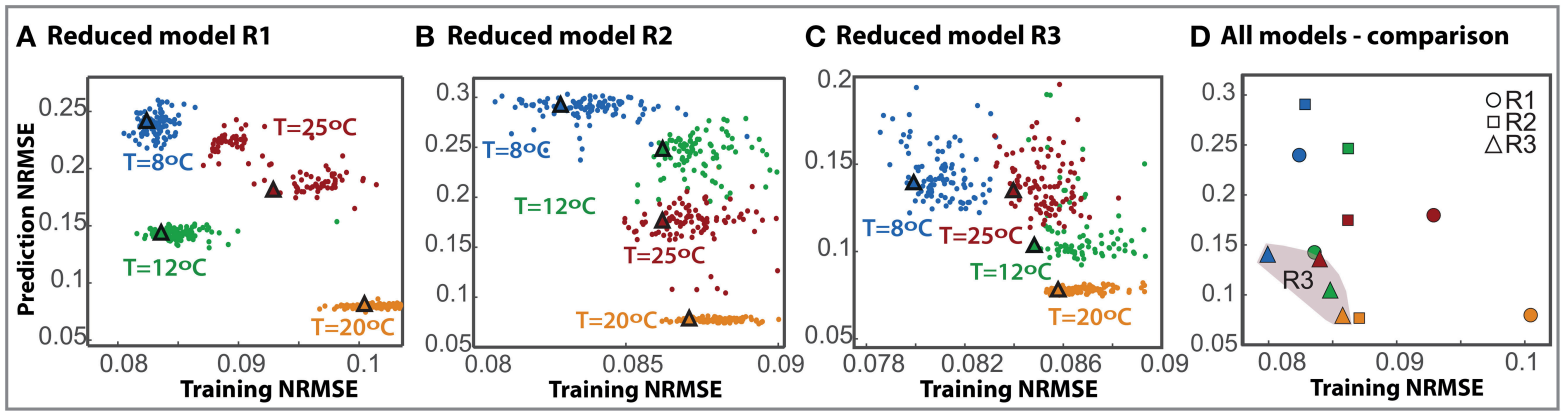
Cross-validation for the selected reduced models. The prediction RMSE is plotted here against the training RMSE for each individual model (dots) and the ensemble (triangle) with different colors owing to different cross-validation scenarios. **(A–C)** Correspond to models R1, R2, and R3 respectively. **(D)** Presents the comparison of all three model ensembles.

To further compare the most successful reduced models we performed *N* = 100 independent parameter estimations from different bootstrapped realizations of the available data to obtain the ensemble of the reduced models. Besides, to test whether reduced models can predict the process out of the training data set we performed a cross-validation analysis. Figures 2A–C show the normalized root mean square error obtained with the training data set vs. the prediction data sets for each reduced model and the corresponding ensemble model (marked with a triangle).

Results demonstrate that the training error is low for all models in all scenarios, between 0.08 − 0.11. The prediction error increases for all models; to a maximum of 0.28 for the second reduced model. As expected, the maximum discrepancy in cross-validation corresponds to extrapolation scenarios for all models.

Remarkably, for model R3 the ensemble solutions, marked with triangles, are more robust than the individual solutions. In fact, in many cases, it is observed that an individual model with a low RMSE value for the training data set does not necessarily perform well in cross-validation. On the contrary, the ensemble is consistent, providing a good compromise between both training and prediction errors.

Figure 2D presents a comparison of the ensembles of all models. R3 model is more robust than the others with the assembles clustered together in the lower error area, NRMSE lower than 0.086 in training and 0.139 in prediction. It should be noted that, despite having less data for the experiments at 20°C, the training NRMSE for the ensemble in cross-validation is only a 7% higher than that obtained for the best case. This result emphasizes the benefits of using multi-experiment data fitting for parameter estimation and cross-validation.

Its consistency, and the associated lower error values, render the ensemble model R3 the best model of those tested to explain and predict cold fermentations by the two species under the specified wine model.

### The best model

**Figure 3:**
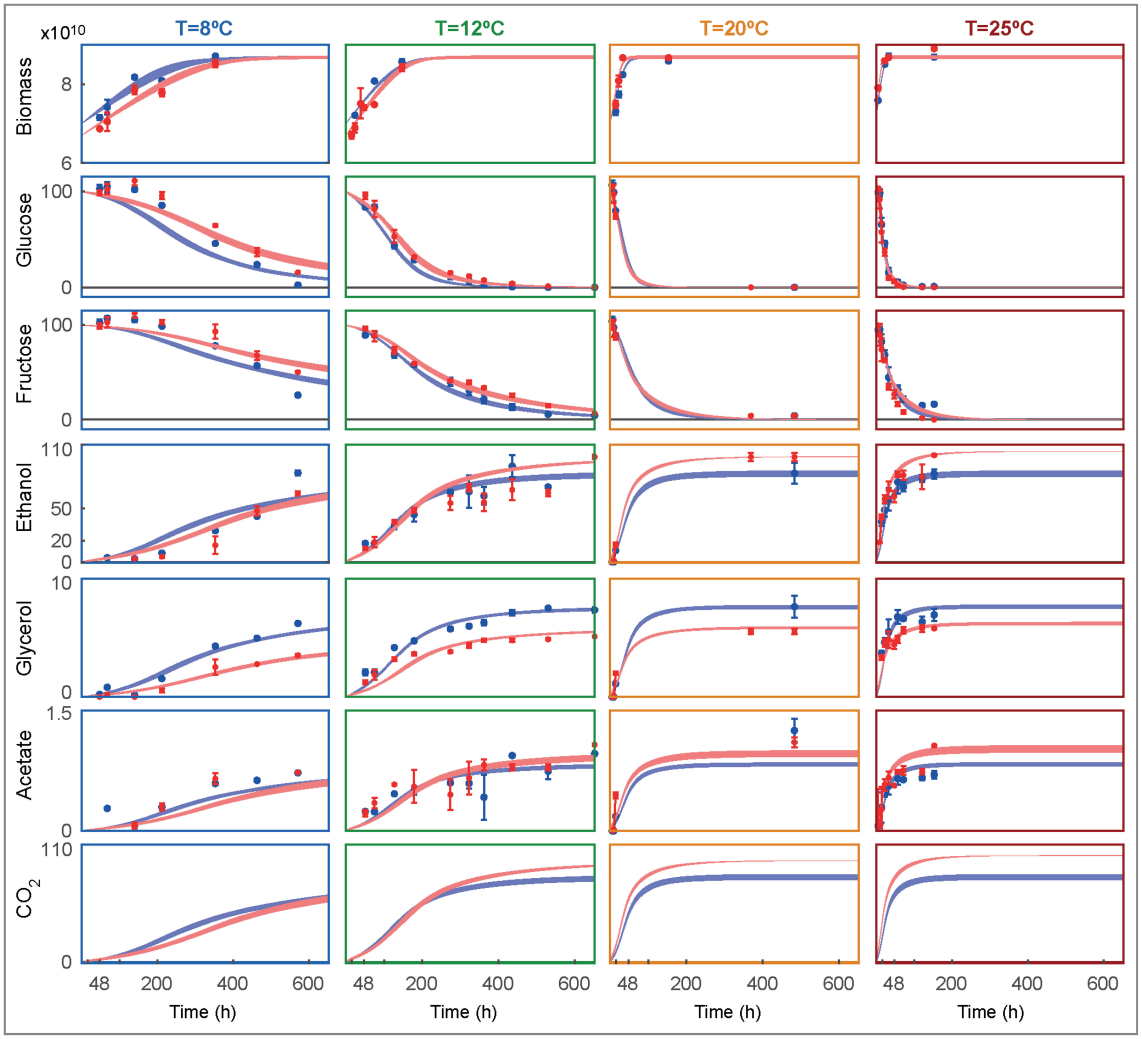
Ensemble of time course predictions for both strains (rows) under different temperatures (columns) as compared to experimental data. The shaded bands depict the predicted non-symmetric 95% confidence interval for SKCR85 (blue) and SCT73 (red). Biomass is shown in decimal logarithm scale (cells/L) while the metabolites are shown in g/L.

Figure 3 shows the experimental data and the ensemble of time course model predictions for both species as obtained for the best ensemble model R3.

The model adequately explains the measurements and the corresponding error bars for both species at all tested temperatures. Temperature affects the duration and rate of alcoholic fermentation as well as final wine quality. At 8°C the system evolves slowly for both species, taking more than 16 days to achieve the maximum biomass. In fact, at 8°C, and after 600 hours the glucose and fructose have not been entirely consumed. In consequence, the production of ethanol and glycerol is significantly lower than the production at higher temperatures. The model fits the glucose and fructose satisfactorily, with the maximum deviations found at the lowest temperature. Both species prefer glucose to fructose, being glucose the first to be consumed in all experimental temperatures. Remarkably, SKCR85 produces less ethanol than SCT73, while producing more glycerol. On the contrary, at 8°C, the production of ethanol is similar in both species, while SKCR85 produces significantly more glycerol, confirming that this species is particularly suited for cold fermentations (Tronchoni et al., 2012).

#### Ensemble of parameters for the selected model

**Figure 4:**
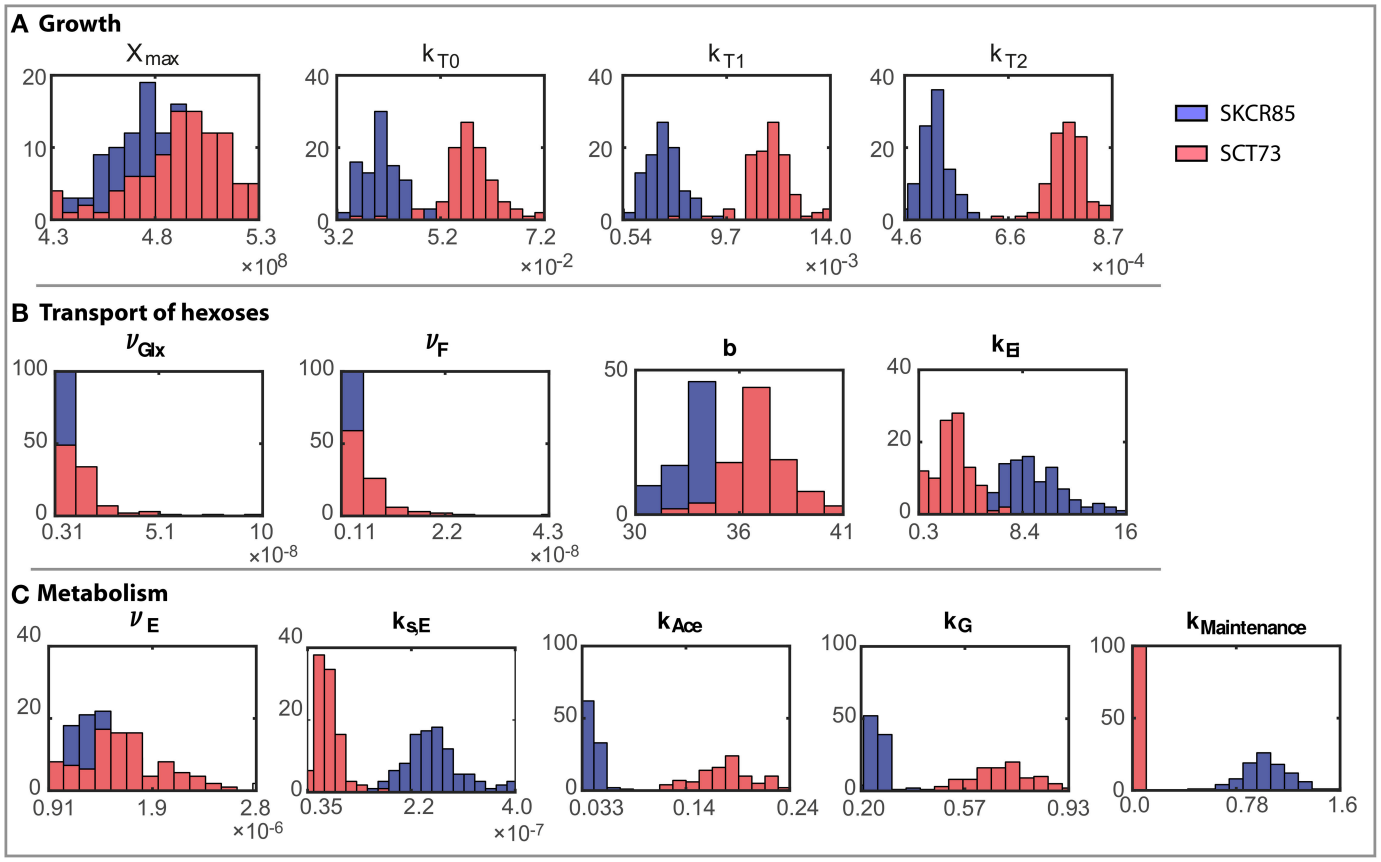
Ensemble of parameter solutions resulting for the multi-experiment data fitting for both strains (model R3). Figures present a comparative analysis of the distributions of parameter values obtained for both species: **(A)** Parameters related to growth, **(B)** Parameters related to transport of hexoses, and **(C)** Parameters related to metabolism. Blue distributions correspond to SKCR85 and red distributions correspond to SCT73.

The ensemble of parameters allows gaining further insights into the mechanisms contributing to the differences observed in the performance of the fermentations mediated by SCT73 and SKCR85. Figure 4 presents the parameter distributions, while Table 2 reports the mean values and the corresponding confidence intervals.

**Table 2:** Mean values of the parameters (θ*) obtained for each strain and the corresponding standard deviation (σ) across the bootstrap estimations.

	**SKCR85**		**SCT73**	
**Parameter name**	θ*	**σ(%)**	θ*	**σ(%)**
*X*_*max*_	4.75 × 10^8^	3.70	4.90 × 10^8^	4.39
*k*_*T*0_	4.05 × 10^−2^	11.10	5.76 × 10^−2^	7.30
*k*_*T*1_	7.14 × 10^−3^	9.56	1.16 × 10^−2^	6.30
*k*_*T*2_	5.14 × 10^−4^	5.13	7.81 × 10^−4^	4.01
*b*	33.5	4.15	36.6	3.81
ν_*Glx*_	4.64 × 10^−9^	17.90	1.70e-08	80.33
ν_*F*_	1.78 × 10^−9^	18.76	6.90 × 10^−9^	80.32
*K*_*E*_*i*__	9.28	25.85	2.93	42.49
*k*_*Ace*_	4.27 × 10^−2^	11.41	1.77 × 10^−1^	15.29
*k*_*E*_	1.41 × 10^−6^	18.32	1.63 × 10^−6^	25.08
*k*_*s, E*_	2.52 × 10^−7^	17.85	6.89e-08	24.99
*k*_*G*_	2.57 × 10^−1^	11.43	7.01 × 10^−1^	14.97
*k*_*Maintenance*_	1.01	17.17	0.00	−

Results reveal that, except for ν_*Glx*_ and ν_*F*_ for SCT73, parameters are computed with high reliability. The mean relative standard deviation corresponds to a 13.35% for those parameters related to SKCR85 and a 14.85% for SCT73. The case of ν_*Glx*_ and ν_*F*_ in SCT73 is particular, since those parameters are highly correlated (See Figure 2 in Supplemental Data) and some outliers appear in the ensemble bootstrap approach due to the large bounds used in parameter estimation.

Parameter values differ substantially for SCT73 and SKCR85, indicating distinct behaviors concerning growth, hexoses transport, and metabolism.

**Figure 5:**
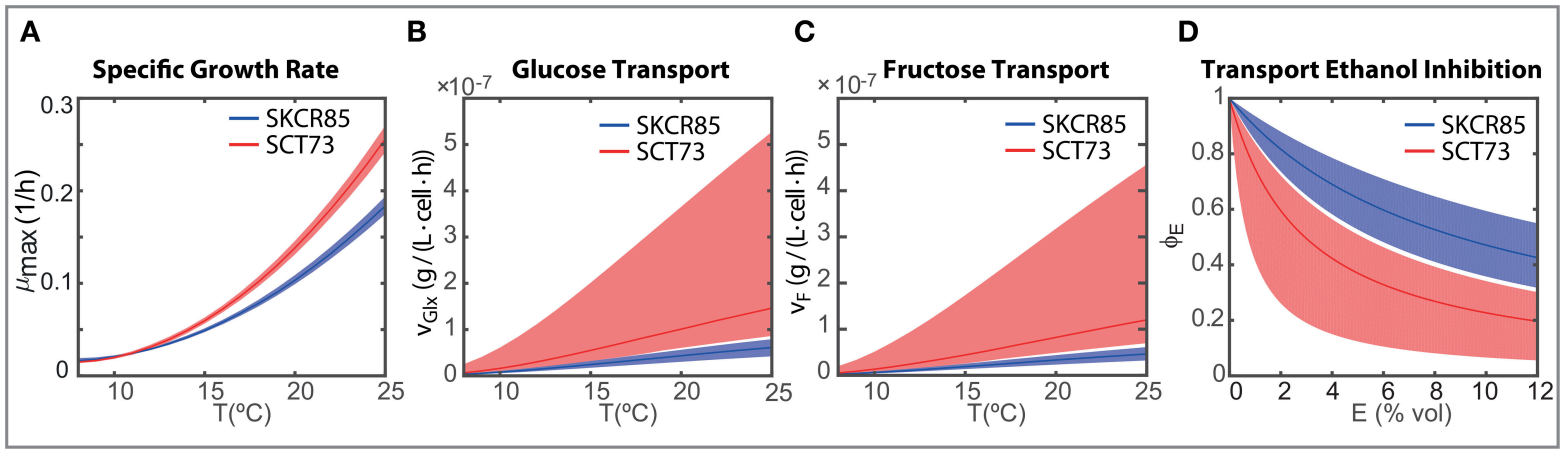
Strain dependent ensemble predictions for **(A)** maximum specific growth rate depending on the temperature; **(B)** temperature dependent glucose transport per cell for initial concentration 100 g/L glucose and 0 g/L ethanol; **(C)** temperature dependent fructose transport per cell for initial concentration 100 g/L fructose and 0 g/L ethanol; **(D)** inhibitory effect of ethanol on the transport of glucose and fructose.

The maximum carrying capacity (*X*_*max*_) is 3% higher for SCT73, meaning that the intra-specific competence is lower for SCT73 than for SKCR85. Temperature and ethanol content strongly affect the specific growth rate. Despite OD_600_ data does not suffice to distinguish temperature and ethanol effects in biomass growth, we can draw some conclusions from the comparative analysis of the specific growth rate for both species (Figure 5A). The cryotolerant SKCR85 and SCT73 grow at similar rates at lower temperatures, between 8 and 12°C; cases in which the maximum ethanol would barely exceed 50 g/L. At higher temperatures, closer to the optimal growth temperature, SCT73 grows around a 40% faster than SKCR85. The fact that at those temperatures ethanol production is high would indicate that SKCR85 is more susceptible to ethanol, which is in agreement with previously published results (Arroyo-López et al., 2010).

Nevertheless, the differences in growth between both species do not explain their distinct fermentation performance. In fact, the differences found in the transport of hexoses play a crucial role (see Figures 5B–D).

In our model, both the temperature and the ethanol affect the transport of hexoses. As mentioned above, both species prefer glucose to fructose (ν_*Glx*_ > ν_*F*_), being glucose the first to be consumed in all experimental temperatures. However, the transport rates vary significantly between species.

Figures 5B,C present the ensemble solutions and the associated uncertainty for the glucose and fructose transport. The uncertainty on the associated parameters explains the uncertainty of the transport activity for SCT73. However, since there is no overlap between the uncertainty intervals between species and the ensemble solutions are clearly distinguishable we are able to perform a fair comparison of the transport activities between species.

The rates of transport of glucose and fructose for SCT73 are around 3.7 the value obtained for SKCR85. This result would confirm that the fitness advantage of *S. cerevisiae* species in fermentation is related to a quicker sugar uptake (Piškur et al., 2006; López-Malo et al., 2013).

Hexoses are carried via facilitated diffusion mediated by the HXT gene family. Different genes show distinct capacities and affinities toward hexoses (see the recent review by Bisson and Walker, 2016). In general, carriers display lower affinities for fructose as compared to glucose (Boles and Hollenberg, 1997), which would explain that ν_*Glx*_ is greater than ν_*F*_ in both species.

Remarkably, Karpel et al. (2008) showed that hexose transporters are distinctly tuned and specialized in *S. cerevisiae* laboratory and wine strains. As for SKCR85, the genetic sequences identities are much lower than between different *S. cerevisiae* strains (data not shown). Our hypothesis is that these lower identities may eventually mean differences in transporters affinity, level and moment of expression during fermentation which would explain the disparity in transport found by the modeling approach.

On the other hand, transport is affected by temperature and ethanol. The intensity of the temperature effect as measured by the parameter *b* differs a 10% between species. These differences have a clear impact on the initial transport of hexoses (when *E* ≈ 0) as illustrated in Figures 5B,C. The Figures show that specially at higher temperatures, SCT73 presents a greater hexose transport per cell. Remark that this is still true despite the variability associated with the transport parameters for SCT73.

The transport of hexoses will vary with time, i.e., as soon as the cells start producing ethanol. Figure 5D shows the inhibition of the transport of hexoses due to the production of ethanol (ϕ_*E*_(*E*)). The ethanol inhibition is driven by the value of *k*_*Ei*_ which is around three times higher in SKCR85 than in SCT73. This difference between the parameter values leads to greater inhibition of the transport in SCT73 than in SKCR85 and the inhibitory effect increases with the amount of ethanol. Our results indicate that transport would be reduced to up to a 20% for SCT73 and 40% for SCR85.

Santos et al. (2008) analyzed how the individual glucose transporters respond to the presence of ethanol, and how the growth phase influenced that response. Their results revealed that all the relevant transporters (HXT1-HXT7), except for HXT2, showed different sensitivities to ethanol as a function of the growth stage. For some strains, they demonstrated that the transporters HXT1 and HXT3 were less sensitive to ethanol in exponential-phase cells than in stationary-phase cells. In contrast, the intermediate- and high-affinity transporters HXT4-HXT7 exhibited a higher inhibition of glucose transport by ethanol in exponential-phase cells than in stationary-phase cells while HXT2 transporter was strongly inhibited in both growth phases. Taking into consideration their results it is plausible that the inhibitory effect gradually increases to achieve its maximum at later stages of the fermentation (stationary phase) when more ethanol is present. Our results indicate that transport would be reduced up to 20% for SCT73 and a 40% for SCR85.

The values of the metabolism-related parameters suggest that SCT73 metabolism is faster than SCR85. While SKCR85 requires directing some hexoses to cellular maintenance, it seems that SCT73 heads practically all hexoses to fermentation products, i.e., contributing to its enhanced fermentative performance. As a consequence, the process characteristic times (for example the time to consume the 90% of hexoses, t_90_) are longer for SKCR85.

Besides, there are substantial differences in the final production of ethanol. SCT73 produces more ethanol than SKCR85, particularly at higher temperatures. This fact may be explained taking into account that SKCR85 directs greater part of the FP6 to produce glycerol as already discussed in the literature (Oliveira et al., 2014) and predicted by the model.

### Optimization of fermentation parameters

**Figure 6:**
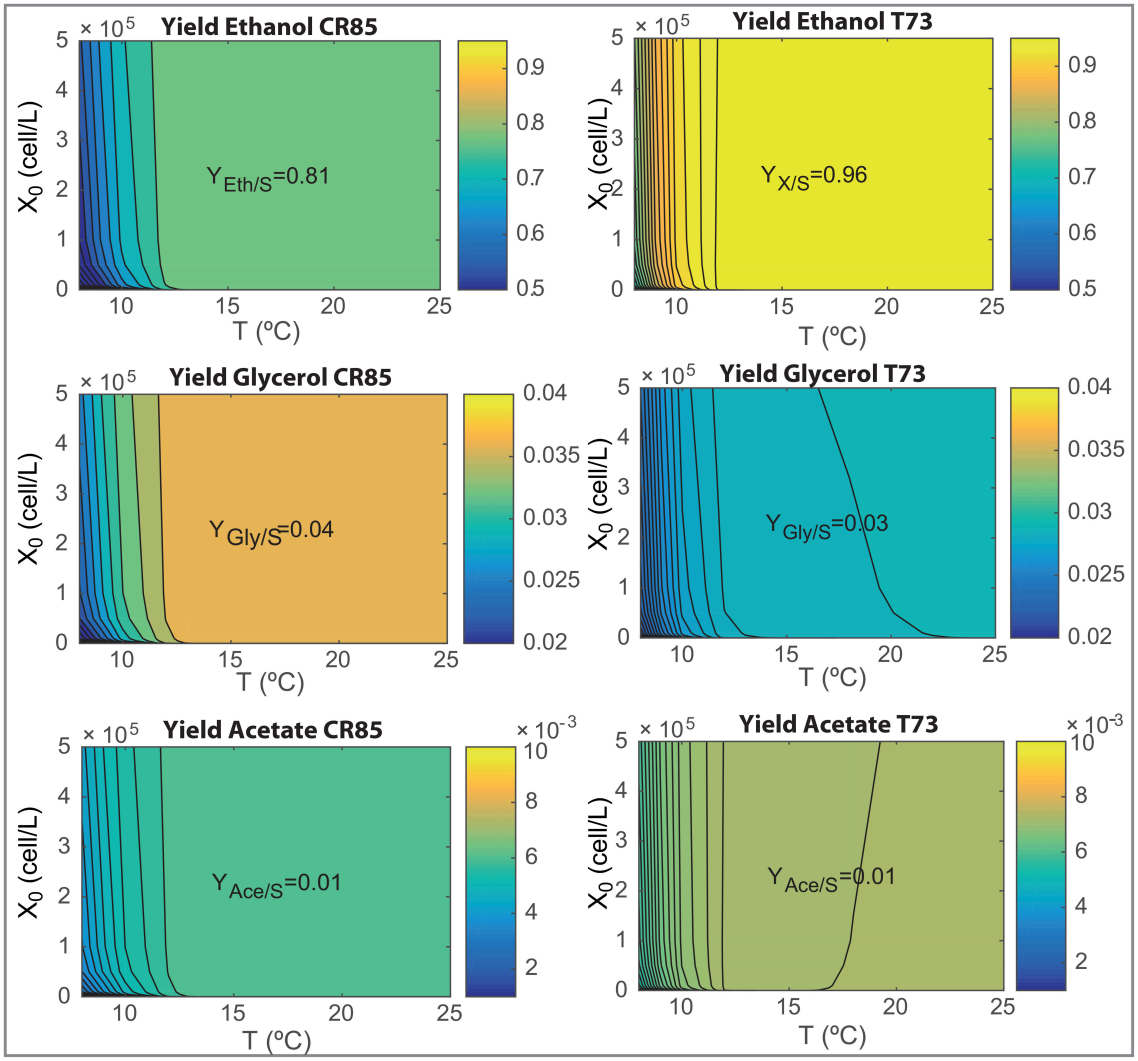
Yields of ethanol, glycerol and acetate as functions of the initial inoculation and the fermentation temperatures for both species as obtained with the ensemble model R3.

**Figure 7:**
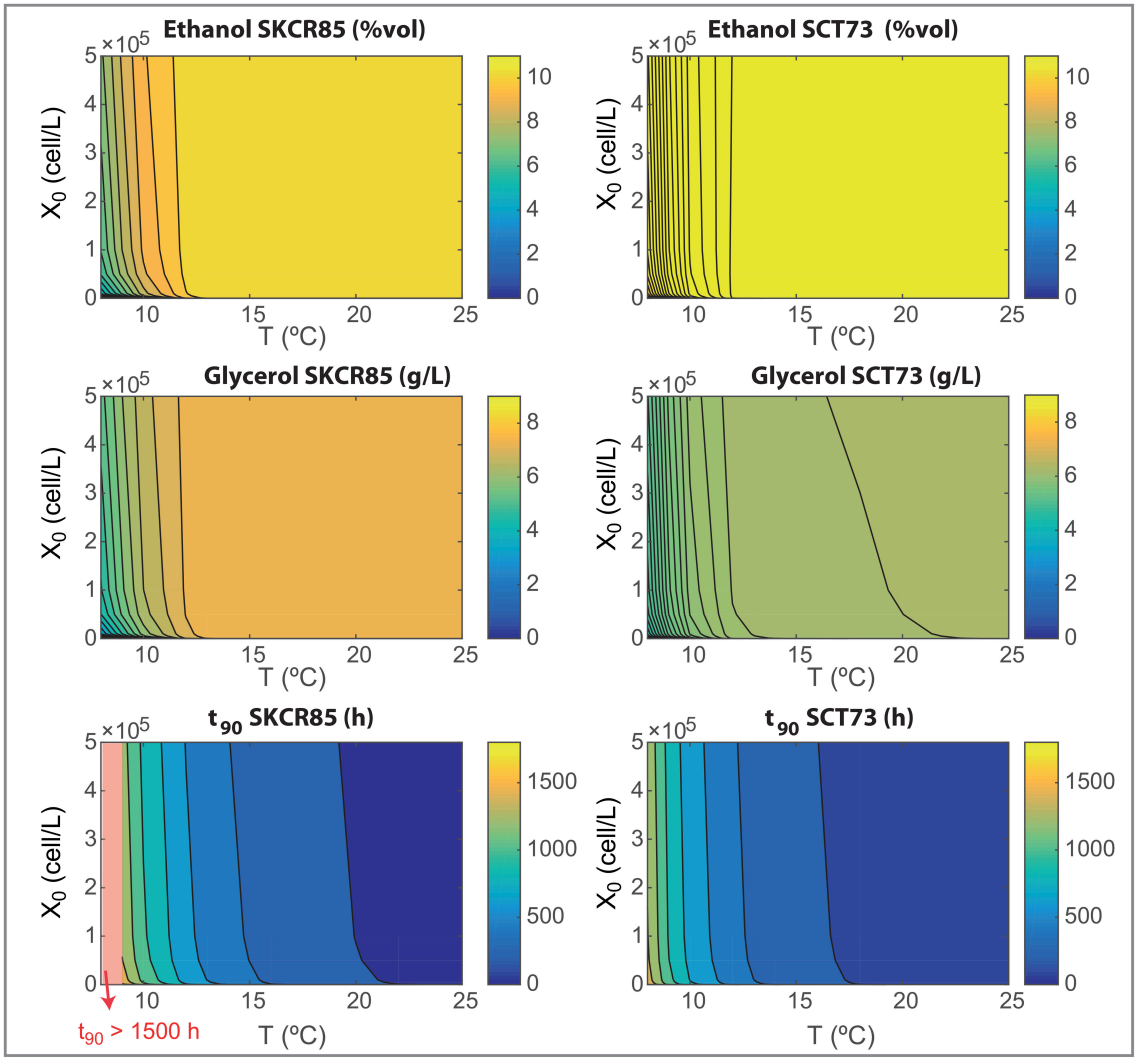
Wine final composition and *t*_90_ as functions of the initial inoculation and the fermentation temperatures for both species as obtained with the ensemble model R3.

The design of novel wine making processes must take into account the final composition of wine as well as the ability for yeast to consume the hexoses present in the must. We now use the model to analyze the effects on processing temperature (in the range, 8–25°C) and initial inoculation (in the range, 0−5·10^5^) on the most relevant fermentation parameters: process yields, final ethanol and glycerol content and the time required to consume the 90% of the initial glucose and fructose content (*t*_90_). Results are shown in Figures 6, 7.

Figure 6 show how SCT73 is substantially more effective in transforming hexoses in ethanol for all tested conditions. The maximum yield corresponds to a 0.96 for SCT73 and 0.81 for SKCR85. Only at very low temperatures the yield for SCT73 reduces to a value similar to the maximum achieved by SKCR85. SKCR85 is more effective than SCT73 yielding glycerol for all conditions tested. SCT73 achieves the maximum glycerol yield at higher temperatures (T>22.5°C) for all inoculations. Similar values can be achieved at around 17°C by increasing the initial inoculation. The yield of acetate is quite insensitive to temperature and initial inoculation, only at very low temperatures (T < 10°C) a slight reduction in yield is observed for both species.

Differences in yields explain the results shown in Figure 7. SKCR85 will produce wines with less ethanol but with higher amounts of glycerol than SCT73 in all tested conditions. Remarkably the production of glycerol is distinctive in SKCR85, it was not possible to achieve the same production of glycerol with SCT73 in any of the conditions tested.

SKCR85 performs similarly, in the sense of final ethanol and glycerol production, in a wide range of temperatures 12.5 − 25°C. Of course, process duration and energy consumption would be different. In contrast, to maximize glycerol content in fermentations driven by SCT73 we would need higher temperatures in the range 18 − 22°C depending on the initial inoculation.

Summing up, the use of SKCR85 will lead to lower ethanol and higher glycerol wines no matter the temperature or the initial inoculation; the best compromise will come from the ethanol/glycerol sought and energy-processing time considerations.

## Conclusions

This work approached the modeling of wine fermentation by two *Saccharomyces* yeast species under different low processing temperatures. We paid major emphasis on achieving a minimal yet robust model. For this purpose we implemented a modeling pipeline which involved the formulation of several candidate models whose parameters were computed by multi-experiment data fitting; models were subsequently reduced and selected attending to the compromise between the quality of fit and the number of parameters (Akaike criterion) as well as their cross-validation properties.

The best model is based on the logistic growth model. The more usual models incorporating the role of substrates inhibition in growth resulted in less robust alternatives due to the poor identifiability of the corresponding parameters. Also, the usual Michaelis-Menten transport formulation could be reduced to a generalized mass action model (linear model) without impacting the quality of the fit and predictive capabilities.

Model predictions were robustified by an ensemble modeling approach. The ensemble satisfactorily predicts process performance thus being suitable for exploring alternative fermentation conditions to optimize final product quality.

We have explored some possibilities by modifying the temperature and initial inoculation. However, more flexibility could be achieved if we also design the feed of hexoses and assimilable nitrogen. This flexibility could be attained by training the models with additional data obtained under various initial hexoses and nitrogen contents. This would allow to either identify an explicit dependency of *N*_*max*_ on substrates or to improve identifiability of other candidate models N1 or N2.

In addition, the somehow complementary performance observed between the two species: higher ethanol production by SCT73 and higher glycerol production by SKCR85, offer even further possibilities to improve the feasibility of low-temperature wine fermentations. Here we explored mono-culture cold fermentations. However, we envision that the optimal design of co-culture based processes may have a tremendous potential for the wine-making industry.

## Author contributions

EB-C and AQ designed the work; DH and EB-C formulated the model; DH performed the optimizations; AQ and JA-d-R conceived and designed the experiments; JA-d-R performed the experiments; all authors analyzed the results and drafted the manuscript.

### Conflict of interest statement

The authors declare that the research was conducted in the absence of any commercial or financial relationships that could be construed as a potential conflict of interest.

## References

[B1] Alonso-del Real J. Lairn-Peris M. Barrio E. Querol A. (2017). Effect of temperature on the prevalence of *Saccharomyces* non *cerevisiae* species against a *S. cerevisiae* wine strain in wine fermentation: competition, physiological fitness, and influence in final wine composition. Front. Microbiol. 8:150. 10.3389/fmicb.2017.00150 28223968 PMC5293751

[B2] Arroyo-López F. N. Salvadó Z. Tronchoni J. Guillamón J. M. Barrio E. Querol A. (2010). Susceptibility and resistance to ethanol in *Saccharomyces* strains isolated from wild and fermentative environments. Yeast 27, 1005–1015. 10.1002/yea.1809 20824889

[B3] Arroyo-López F. N. Orli S. Querol A. Barrio E. (2009). Effects of temperature, ph and sugar concentration on the growth parameters of *Saccharomyces cerevisiae, S. kudriavzevii* and their interspecific hybrid. Int. J. Food Microbiol. 131, 120–127. 10.1016/j.ijfoodmicro.2009.01.035 19246112

[B4] Balsa-Canto E. Henriques D. Gabor A. Banga J. (2016). AMIGO2, a toolbox for dynamic modeling, optimization and control in systems biology. Bioinformatics 32, 3357–3359. 10.1093/bioinformatics/btw411 27378288 PMC5079478

[B5] Banga J. Balsa-Canto E. Moles C. Alonso A. (2005). Dynamic optimization of bioprocesses: Efficient and robust numerical strategies. J. Biotechnol. 117, 407–419. 10.1016/j.jbiotec.2005.02.013 15888349

[B6] Baranyi J. Roberts T. (1994). A dynamic approach to predicting bacterial growth in food. Int. J. Food Microbiol. 23, 277–294. 10.1016/0168-1605(94)90157-0 7873331

[B7] Berthels N. Cordero Otero R. Bauer F. Thevelein J. Pretorius I. (2004). Discrepancy in glucose and fructose utilisation during fermentation by *Saccharomyces cerevisiae* wine yeast strains. FEMS Yeast Res. 4, 683–689. 10.1016/j.femsyr.2004.02.005 15093771

[B8] Bisson L. F. Fan Q. Walker G. (2016). Sugar and glycerol transport in *Saccharomyces* cerevisiae. Adv. Exp. Med. Biol. 892, 125–168. 10.1007/978-3-319-25304-6_6 26721273

[B9] Boles E. Hollenberg C. P. (1997). The molecular genetics of hexose transport in yeasts. FEMS Microbiol. Rev. 21, 85–111. 10.1111/j.1574-6976.1997.tb00346.x 9299703

[B10] Boulton R. (1980). The prediction of fermentation behavior by a kinetic model. Am. J. Enol. Vitic. 31, 40–46.

[B11] Breiman L. (1996). Bagging predictors. Mach. Learn. 24, 123–140. 10.1007/BF00058655

[B12] Bühlmann P. Yu B. (2002). Analyzing bagging. Ann. Stat. 30, 927–961. 10.1214/aos/1031689014

[B13] Bühlmann P. (2012). Bagging, boosting and ensemble methods, in Handbook of Computational Statistics (Springer), 985–1022.

[B14] Burnham K. Anderson D. (2002). Model Selection and Multimodel nference: A Practical Information-Theoretic Approach, 2nd Edn. (New York, NY: Springer-Verlag).

[B15] Caro I. Prez L. Cantero D. (1991). Development of a kinetic model for the alcoholic fermentation of must. Biotechnol. Bioeng. 38, 742–748. 10.1002/bit.260380708 18600800

[B16] Charnomordic B. David R. Dochain D. Hilgert N. Mouret J.-R. Sablayrolles J.-M. (2010). Two modelling approaches of winemaking: first principle and metabolic engineering. Math. Comp. Model Dyn. 16, 535–553. 10.1080/13873954.2010.514701

[B17] Chis O. Villaverde A. Banga J. Balsa-Canto E. (2016). On the relationship between sloppiness and identifiability. Math. Biosci. 282, 147–161. 10.1016/j.mbs.2016.10.009 27789352

[B18] Ciani M. Morales P. Comitini F. Tronchoni J. Canonico L. Curiel J.. (2016). Non-conventional yeast species for lowering ethanol content of wines. Front. Microbiol. 7:642. 10.3389/fmicb.2016.00642 27199967 PMC4854890

[B19] Coleman M. Fish R. Block D. (2007). Temperature-dependent kinetic model for nitrogen-limited wine fermentation. Appl. Environ. Microbiol. 73, 5875–5884. 10.1128/AEM.00670-07 17616615 PMC2074923

[B20] Cramer A. Vlassides S. Block D. E. (2002). Kinetic model for nitrogen-limited wine fermentations. Biotechnol. Bioeng. 77, 49–60. 10.1002/bit.10133 11745173

[B21] David R. Dochain D. Mouret J.-R. Vande Wouwer A. Sablayrolles J.-M. (2010). Dynamical modeling of alcoholic fermentation and its link with nitrogen consumption, in IFAC Proceedings Volumes 43:6 (Leuven), 496–501.

[B22] Efron B. Tibshirani R. (1988). An Introduction to the Bootstrap. New York, NY: Chapman & Hall.

[B23] Egea J. A. Vazquez E. Banga J. R. Marti R. (2009). Improved scatter search for the global optimization of computationally expensive dynamic models. J. Global Opt. 43, 175–190. 10.1007/s10898-007-9172-y

[B24] Elsner J. Schmertmann C. (1994). Assessing forecast skill through cross validation. Weather Forecast 9, 619–624. 10.1175/1520-0434(1994)009<0619:AFSTCV>2.0.CO;2

[B25] Henriques D. Villaverde A. Rocha M. Saez-Rodriguez J. Banga J. (2017). Data-driven reverse engineering of signaling pathways using ensembles of dynamic models. PLoS Comp. Biol. 13:e1005379. 10.1371/journal.pcbi.1005379 28166222 PMC5319798

[B26] Hindmarsh A. Brown P. Grant K. Lee S. L. Serban R. Shumaker D. E. (2005). SUNDIALS: suite of nonlinear and differential/algebraic equation solvers. ACM Trans. Math. Softw. 31, 363–396. 10.1145/1089014.1089020

[B27] Hjersted J. Henson M. Mahadevan R. (2007). Genomescale analysis of *Saccharomyces cerevisiae* metabolism and ethanol production in fedbatch culture. Biotechnol. Bioeng. 97, 1190–1204. 10.1002/bit.21332 17243146

[B28] Karpel J. Place W. Bisson L. (2008). Analysis of the major hexose transporter genes in wine strains of *Saccharomyces cerevisiae*. Am. J. Enol. Vitic. 59, 265–275. Available online at: http://www.ajevonline.org/content/59/3/265

[B29] Leão C. Van Uden N. (1982). Effects of ethanol and other alkanols on the glucose transport system of *SSaccharomyces cerevisiae*. Biotechnol. Bioeng. 24, 2601–2604. 10.1002/bit.260241124 18546229

[B30] López-Malo M. Querol A. Guillamón J. M. (2013). Metabolomic comparison of *Saccharomyces cerevisiae* and the cryotolerant species *S. bayanus* var. *uvarum* and *S. kudriavzevii* during wine fermentation at low temperature. PLOS ONE 8:60135. 10.1371/journal.pone.0060135 23527304 PMC3603904

[B31] Malherbe S. Fromion V. Hilgert N. Sablayrolles J.-M. (2004). Modeling the effects of assimilable nitrogen and temperature on fermentation kinetics in enological conditions. Biotechnol. Bioeng. 86, 261–272. 10.1002/bit.20075 15083506

[B32] Marín R. (1999). Alcoholic fermentation modelling: Current state and perspectives. Am. J. Enol. and Vitic 50, 166–178.

[B33] Oliveira B. Barrio E. Querol A. Pérez-Torrado R. (2014). Enhanced enzymatic activity of glycerol-3-phosphate dehydrogenase from the cryophilic *Saccharomyces kudriavzevii*. PloS ONE 9:e87290. 10.1371/journal.pone.0087290 24498063 PMC3907487

[B34] Pérez-Torrado R. Barrio E. Querol A. (2017). Alternative yeasts for winemaking: *Saccharomyces* non-*cerevisiae* and its hybrids. Crit. Rev. Food Sci. Nut. [Epub ahead of print]. 10.1080/10408398.2017.1285751 28362111

[B35] Pérez-Torrado R. Oliveira B. Zemancikova J. H. S. A. Q. (2016). Alternative glycerol balance strategies among *Saccharomyces* species in response to winemaking stress. Front. Microbiol. 7:435. 10.3389/fmicb.2016.00435 27064588 PMC4814467

[B36] Piškur J. Rozpedowska E. Polakova S. Merico A. Compagno C. (2006). How did *Saccharomyces* evolve to become a good brewer? Trends Genetics 22, 183–186. 10.1016/j.tig.2006.02.002 16499989

[B37] Pizarro F. Vargas F. A. Agosin E. (2007). A systems biology perspective of wine fermentations. Yeast 24, 977–991. 10.1002/yea.1545 17899563

[B38] Postmus J. Canelas A. B. Bouwman J. Bakker B. van Gulik W. de Mattos M. J. T.. (2008). Quantitative analysis of the high temperature-induced glycolytic flux increase in *Saccharomyces cerevisiae* reveals dominant metabolic regulation. J. Biol. Chem. 283, 23524–23532. 10.1074/jbc.M802908200 18562308 PMC3259761

[B39] Rossignol T. Dulau L. Julien A. Blondin B. (2003). Genome-wide monitoring of wine yeast gene expression during alcoholic fermentation. Yeast 20, 1369–1385. 10.1002/yea.1046 14663829

[B40] Sainz J. Pizarro F. Pérez-Correa J. R. Agosin E. (2003). Modeling of yeast metabolism and process dynamics in batch fermentation. Biotechnol. Bioeng. 81, 818–828. 10.1002/bit.10535 12557315

[B41] Salvadó Z. Arroyo-López F. Barrio E. Querol A. Guillamón J. (2011). Quantifying the individual effects of ethanol and temperature on the fitness advantage of *Saccharomyces cerevisiae*. Food Microbiol. 28, 1155–1161. 10.1016/j.fm.2011.03.008 21645814

[B42] Santos J. Sousa M. Cardoso H. Inácio J. Silva S. Spencer-Martins I.. (2008). Ethanol tolerance of sugar transport, and the rectification of stuck wine fermentations. Microbiology 154, 422–430. 10.1099/mic.0.2007/011445-0 18227246

[B43] Stribny J. Querol A. Pérez-Torrado R. (2016). Differences in enzymatic properties of the *Saccharomyces kudriavzevii* and *Saccharomyces uvarum* alcohol acetyltransferases and their impact on aroma-active compounds production. Front. Microbiol. 7:897. 10.3389/fmicb.2016.00897 27375606 PMC4894917

[B44] Tai S. Daran-Lapujade P. Luttik M. Walsh M. Diderich J. Krijger G.. (2007). Control of the glycolytic glux in *Saccharomyces cerevisiae* grown at low temperature: a multi-level analysis in anaerobic chemostat cultures. J. Biol. Chem. 282, 10243–10251. 10.1074/jbc.M610845200 17251183

[B45] Tashman L. (2000). Out-of-sample tests of forecasting accuracy: an analysis and review. Int. J. Forecast 16, 437–450. 10.1016/S0169-2070(00)00065-0

[B46] Teusink B. Diderich J. Westerhoff H. Van Dam K. Walsh M. (1998). Intracellular glucose concentration in derepressed yeast cells consuming glucose is high enough to reduce the glucose transport rate by 50%. J. Bacteriol. 180, 556–562. 9457857 10.1128/jb.180.3.556-562.1998 PMC106921

[B47] Tronchoni J. Rozès N. Querol A. Guillamón J. (2012). Lipid composition of wine strains of *Saccharomyces kudriavzevii* and *Saccharomyces cerevisiae* grown at low temperature. Int. J. Food Microbiol. 155, 191–198. 10.1016/j.ijfoodmicro.2012.02.004 22405355

[B48] Varela C. Pizarro F. Agosin E. (2004). Biomass content governs fermentation rate in nitrogen-deficient wine musts. App. Environ. Microbiol. 70, 3392–3400. 10.1128/AEM.70.6.3392-3400.2004 15184136 PMC427798

[B49] Vargas F. A. Pizarro F. Pérez-Correa J. R. Agosin E. (2011). Expanding a dynamic flux balance model of yeast fermentation to genome-scale. BMC Syst. Biol. 5:75. 10.1186/1752-0509-5-75 21595919 PMC3118138

[B50] Vilas C. Arias-Mndez A. Garcia M. Alonso A. Balsa-Canto E. (2018). Towards predictive food process models: a protocol for parameter estimation. Crit. Rev. Food Sci. Nut. 58, 436–449. 10.1080/10408398.2016.1186591 27246577

[B51] Walter E. Pronzato L. (1997). Identification of Parametric Models from Experimental Data. Gateshead: Springer.

