# Supplementary material for: Saccharomyces cerevisiae and S. kudriavzevii Synthetic Wine Fermentation Performance Dissected by Predictive Modeling
Source: Front Microbiol. 2018 Feb 2;9:88. doi: 10.3389/fmicb.2018.00088 (PMC5801724; doi:10.3389/fmicb.2018.00088)
Supplement: Supplementary file 1 [file Presentation1.pdf]

Supplementary materials: *Saccharomyces cerevisiae* and *S. kudriavzevii* synthetic wine fermentation performance dissected by predictive modeling

D. Henriques, J. Alonso-del-Real, A. Querol and E. Balsa-Canto

[1] *(Bio)process engineering group, IIM-CSIC, Vigo, Spain*
[2] *Grupo de Biología de Sistemas en Levaduras de Interés Biotecnológico, IATA-CSIC, Valencia, Spain*

Correspondence*:
Eva Balsa-Canto
(Bio)process engineering group, IIM-CSIC, C/Eduardo Cabello N6, 36208-Vigo, Spain, ebalsa@iim.csic.es

S1. Model selection and reduction

Modeling was approached from a systems identification perspective including the following steps: formulation of candidate models, multi-experiment parameter estimation, model selection and reduction, ensemble modeling and cross-validation.

We formulated several candidate models which account for the relevant process variables (biomass growth, sugars, ethanol, glycerol, acetate) based on different mechanisms described in literature. Exact formulations are described in the Main text.

All candidate models consist of a set of ordinary differential equations whose solution depends on the given initial conditions, process temperature and the value of a number of unknown parameters.

A multi-experiment data fitting approach was applied to compute unknown adjustable parameters for all models. Results are summarized in the sequel.

Tables include the step reduction number, the least squares value achieved, the number of data used, the number of unknown parameters, the Akaike criterion value and the name of the file including the model in the supplementary code distribution.

S.1.1. Models N1, R1 and intermediates

Step 1 (Accept): Initial model.

Step 2 (Reject): We assume the transport of glucose and fructose are affected equally by temperature. Two parameters from the ϕ_T expression (one per strain) are removed. In this model the impact is big and AIC increases. Move back to step 1.

Step 3 (Reject): We assume glucose and fructose and glucose are affected equally by ethanol. Two parameters from the ϕ_E expression (one per strain) are removed. In this model the impact is big and AIC increases. Move back to step 1.

Step	RSS	#Data	#Pars	AIC	File
1	6.92	329	31	338.52	MODEL7.csv
2	7.41	329	29	344.29	MODEL21.csv
3	7.80	329	29	351.60	MODEL22.csv
4	6.92	329	27	330.52	MODEL23.csv
5	6.92	329	25	326.52	MODEL24.csv

Table 1. Path followed in the iterative procedure to reduce model N1 to R1 based on the AIC. Step: step reduction number, RSS: best least squares value, #Data: number of data,#Pars: number of unknown parameters, AIC: Akaike criterion value at optimum and File: name of the file including the model in the supplementary code distribution.

Step 4 (Accept): We assume the affinity constants form the Michaelis Menten type kinetics used to describe the transport are unnecessary to describe transport in the operating range. Thus we reduce the model to mass action type kinetics. The AIC improves. The model is accepted. Four parameters are eliminated, two affinity constants (glucose and fructose) per strains.

Step 5 (Accept): We assume mass action type kinetics is sufficient to describe ethanol production ($\upsilon_{\mathrm{F6P}\rightarrow\mathrm{E}}$) in the operating range of the model. Two affinity constants are removed (one per strain).

S.1.2. Models N2, R2 and intermediates

Step	RSS	#Data	#Pars	AIC	File
1	5.51	329	35	313.95	MODEL8.csv
2	5.57	329	29	303.41	MODEL9.csv
3	5.57	329	25	295.52	MODEL10.csv
4	5.73	329	23	295.59	MODEL11.csv
5	5.73	329	21	291.58	MODEL13.csv

Table 2. Path followed in the iterative procedure to reduce the model N2 to R2 based on the AIC. Step: step reduction number, RSS: best least squares value, #Data: number of data,#Pars: number of unknown parameters, AIC: Akaike criterion value at optimum and File: name of the file including the model in the supplementary code distribution.

Step 1 (Accept) : Initial model.

Step 2 (Accept) : We assume the transport of glucose and fructose are affected equally by temperature. Six parameters from the ϕ_T expression (3 per strain) are removed.

Step 3 (Accept) : We assume $\phi_{T,A}$, with constant nitrogen data, is able to explain the temperature effect on the transport of hexoses without the need of ν_H.

Step 4 (Accept) : We assume mass action type kinetics is sufficient to describe ethanol production ($\upsilon_{\mathrm{F6P}\rightarrow\mathrm{E}}$) in the operating range of the model. Two affinity constants are removed (one per strain).

Step 5 (Accept: We assume mass action type kinetics is sufficient to describe ethanol production ($\upsilon_{\mathrm{F6P}\rightarrow\mathrm{E}}$) in the operating range of the model. Two affinity constants are removed (one per strain).

S.1.3. Models N3, R3 and intermediates

Step 1 (Accept) : Initial model.

Step 2 (Accept) : We assume the transport of glucose and fructose are affected equally by temperature. Two parameters from the ϕ_T expression (one per strain) are removed.

Step	RSS	#Data	#Pars	AIC	File
1	4.68	329	33	286.65	MODEL15.csv
2	4.73	329	31	284.32	MODEL16.csv
3	4.86	329	27	280.05	MODEL17.csv
4	4.87	329	25	276.26	MODEL18.csv
5	5.15	329	23	280.27	MODEL20.csv

Table 3. Path followed in the iterative procedure to reduce model N3 to R3 based on the AIC. Step: step reduction number, RSS: best least squares value, #Data: number of data,#Pars: number of unknown parameters, AIC: Akaike criterion value at optimum and File: name of the file including the model in the supplementary code distribution.

Step 3 (Accept) : We assume the affinity constants form the Michaelis Menten type kinetics used to describe the transport are unnecessary to describe transport in the operating range. Thus we reduce the model to mass action type kinetics. The AIC improves. The model is accepted. Four parameters are eliminated, two affinity constants (glucose and fructose) per strains.

Step 4 (Accept) : We assume glucose and fructose and glucose are affected equally by ethanol. Two parameters from the ϕ_E expression (one per strain) are removed.

Step 5 (Reject) : We assume mass action type kinetics is sufficient to describe ethanol production ($v_{\mathrm{F6P}\rightarrow\mathrm{E}}$) in the operating range of the model. Two affinity constants are removed (one per strain). In this model the impact is big and AIC increases. Move back to step 4.

S2. Ensemble of parameter values for the reduced model R3

Following figures present the ensemble of parameters as normalized to their mean values for the sake of comparing their relative confidence intervals.

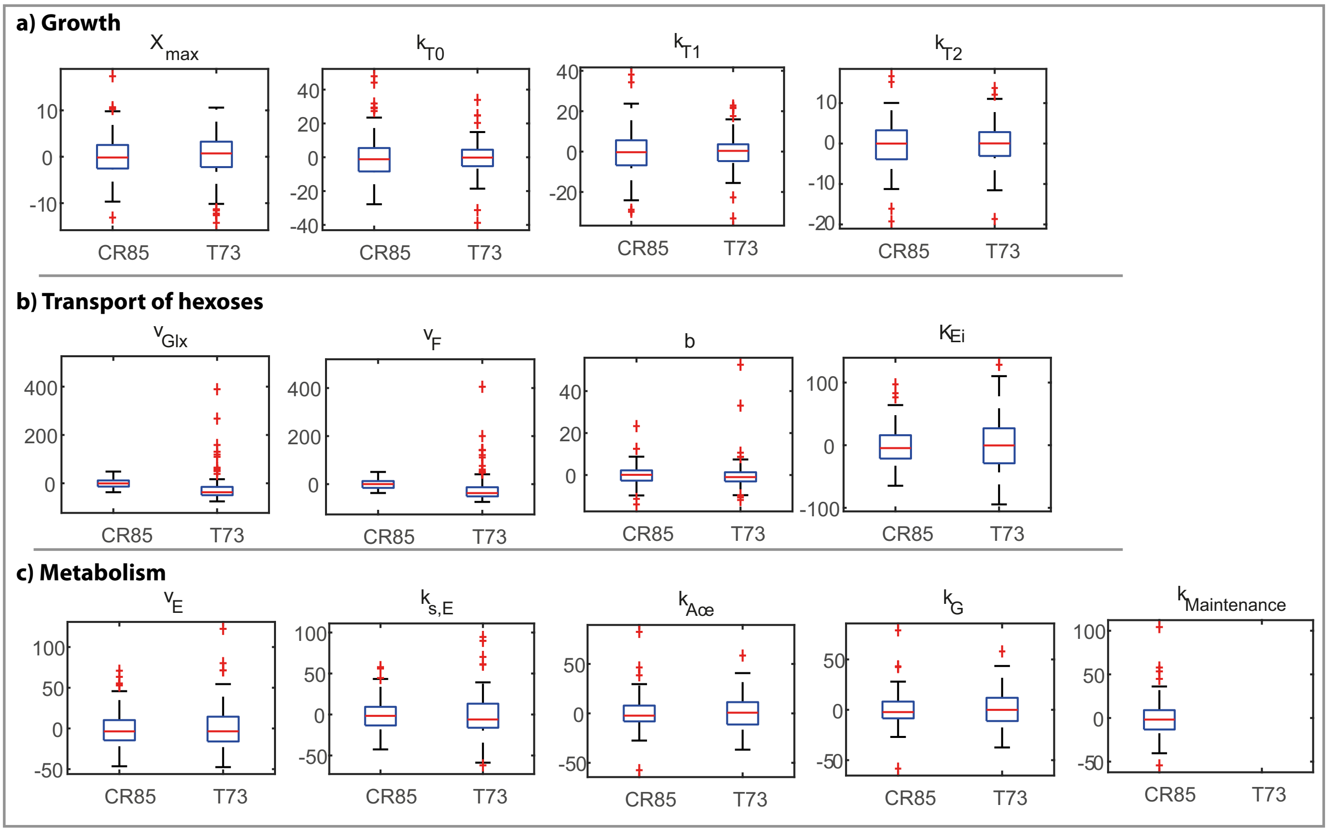

Figure 1. Ensemble of parameters expressed in % relative difference with respect to the mean value: a) Parameters related to growth, b) Parameters related to hexoses transport and d) Parameters related to metabolism.

S3. Correlation analysis of ensemble reduced model R3

The following figure presents the correlation as computed by pairs of parameters. Results reveal that several pairs of parameters are rather correlated ($C\|r_{ij}\| > 0.9$). This is the case of the parameters that define specific growth rate as a function of temperature $\mu(T)$; and parameters related to transport ν_{Glx} and ν_F. The correlation among ν_{Glx} and ν_F translates into significant confidence intervals for both parameters since their bounds could not be precisely estimated from the data.

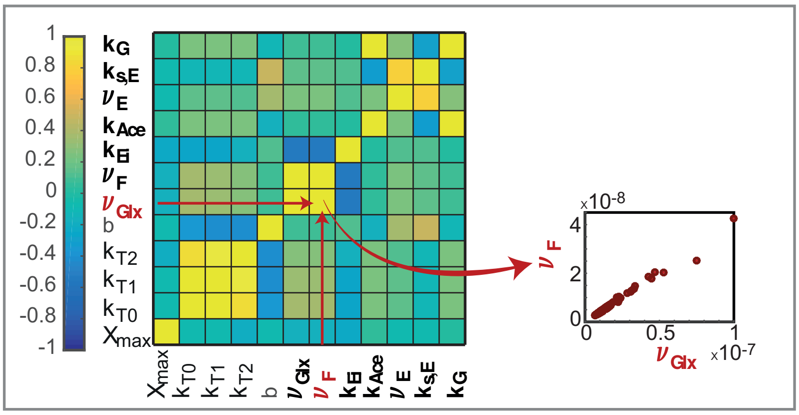

Figure 2. Parametric correlation matrix as evaluated for SCT73. The figure shows the correlation between pairs of parameters, and illustrates the almost linear correlation between ν_{Glx} and ν_F.

S4. Code to reproduce the computational analysis

Model files, experimental data and scripts to reproduce results can be found in a public repository: `http://doi.org/10.5281/zenodo.1115605`.
